# Medication Rules in Herbal Medicine for Mild Cognitive Impairment: A Network Pharmacology and Data Mining Study

**DOI:** 10.1155/2022/2478940

**Published:** 2022-05-18

**Authors:** Z. Chang, Yu-Chun Wang, Danfeng Tian, Wen-Yue Hu, Zhen-Yi Wang, Gan-Lu Liu, Hua-Ping Ma, Yu-Li Hu, Bin Wu, Zhen-Yun Han

**Affiliations:** ^1^Dongfang Hospital of Beijing University of Chinese Medicine, Beijing 100000, China; ^2^Beijing University of Chinese Medicine, Beijing 100000, China; ^3^Shenzhen Hospital of Beijing University of Chinese Medicine, Shenzhen 518000, Guangdong, China

## Abstract

**Background:**

Although traditional Chinese medicine (TCM) has good efficacy in the treatment of mild cognitive impairment (MCI), especially memory improvement and safety, its substance basis and intervention mechanism are particularly complex and unknown. Therefore, based on network pharmacology and data mining, this study aims to explore the rules, active ingredients and mechanism of TCM in the treatment of MCI.

**Methods:**

By searching the GeneCard, OMIM, DisGeNET and DrugBank databases, we obtained the critical targets associated with MCI. We matched the components and herbs corresponding to the important targets in the TCMSP platform. Using Cytoscape 3.7.2 software, we constructed a target-component-herb network and conducted a network topology analysis to obtain the core components and herbs. Molecular docking was used to preliminarily analyze and predict the binding activities and main binding combinations of the core targets and components. Based on the analysis of the properties, flavor and meridian distribution of herbs, the rules of herbal therapy for MCI were summarized.

**Results:**

Twenty-eight critical targets were obtained after the screening. Using the TCMSP platform, 492 components were obtained. After standardization, we obtained 387 herbs. Based on the target-composition-herb network analysis, the core targets were ADRB2, ADRA1B, DPP4, ACHE and ADRA1D. According to the screening, the core ingredients were beta-sitosterol, quercetin, kaempferol, stigmasterol and luteolin. The core herbs were matched to Danshen, Yanhusuo, Gancao, Gouteng and Jiangxiang. It was found that the herbs were mainly warm in nature, pungent in taste and liver and lung in meridian. The molecular docking results showed that most core components exhibited strong binding activity to the target combination regardless of the in or out of network combination.

**Conclusion:**

The results of this study indicate that herbs have great potential in the treatment of MCI. This study provides a reference and basis for clinical application, experimental research and new drug development of herbal therapy for MCI.

## 1. Introduction 

In 1997, Petersen in the United States first proposed the diagnostic standard of mild cognitive impairment (MCI), which is mismatched between memory loss and normal aging but relatively reserved in other cognitive functional domains [1]. This standard is mainly used to describe MCI types that eventually transform into AD clinically [2]. In 2011, the National Institute on Ageing-Alzheimer's Association Task Force proposed new guidelines stating that MCI, as the second stage in the course of AD, should be classified under the AD concept and not as a separate disease [3]. Currently, mild cognitive impairment, which is a transitional condition between normal aging and dementia, is characterized by a progressive decline in memory and other cognitive functions accompanied by a slight impairment in the instrumental living ability [2, 4]. In terms of epidemiology, a systematic review of 123,766 patients showed that the prevalence of MCI among the elderly in China was approximately 15.4%, which varied depending on the demographics, lifestyle, morbidity, screening tools and diagnostic criteria [5]. MCI is an important risk factor for dementia. Studies have shown that approximately 10 to 15 percent of MCI patients progress to dementia each year compared with 1 to 2 percent in the normal elderly population and age-matched controls [6, 7]. The risk of AD in MCI patients was 3.17 times higher than that in the normal elderly, and 5-year and 10-year MCI follow-up studies showed that the probability of MCI converting to AD was 33% and 55.5%, respectively [8, 9]. Therefore, for MCI, early intervention is an important way to effectively delay or block cognitive decline and prevent the occurrence of dementia.

MCI interventions aim to prevent, delay or even reverse the process of AD transformation. According to the latest international MCI guidelines, currently, no drugs or foods are approved by the Food and Drug Administration (FDA) for the treatment of MCI [10–12]. Additionally, relevant evidence-based studies have shown that cholinesterase inhibitors do not lead to significant improvement in cognitive function in MCI; in contrast, the early application of cholinesterase inhibitors may cause cognitive impairment [13, 14]. Traditional Chinese medicine (TCM) has a long history of treating memory loss. Pharmacological studies and clinical trials have confirmed that TCM, including single drugs and compound TCM preparations, have a positive effect on improving cognitive impairment and enhancing memory function [15, 16]. For example, in a 24-month randomized placebo-controlled study, Bushen capsules, a Chinese herb compound, improved multiple cognitive domains and increased functional local and global connectivity in the right precuneus default mode network [17]. In patients with amnestic mild cognitive impairment, Qinggongshoutao (QGST) pill had significant benefits in improving the overall cognitive ability and reducing the rate of progression of Alzheimer's disease. Relevant review studies have shown that some active extracts or components of Chinese herbs, such as ginseng, Polygala, Schisandra sphenanthera, Andrographis paniculata, Gynostemmae pentaphylli herba and Lycium barbarum, can prevent and treat AD by reducing A*β* production, inhibiting cell apoptosis, inhibiting neuroinflammation, regulating autophagy, resisting oxidative stress and improving mitochondrial dysfunction [18, 19].

**Figure 1:**
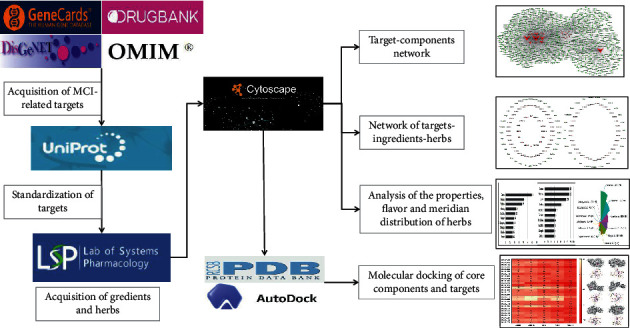
The overall flow chart of this study based on network pharmacology and data mining.

Network pharmacology is a new analysis that reveals the multidimensional mechanism between drugs and diseases by constructing and analyzing the complex network of “drug-gene–target-disease” from the perspective of the system level and biological network overall [20–22]. This approach is widely used in the discovery of drugs and active compounds of TCM, the interpretation of the mechanism of action of traditional Chinese medicine compounds, the compatibility of prescriptions, etc. [23]. Network pharmacology aims to explore the interaction between drugs and the body from a systematic and holistic perspective, which is consistent with the characteristics of integration and systematization and consideration of the interaction of TCM [24]. Network pharmacology not only provides new ideas for the study of the complex systems of TCM but also provides a new method for rational clinical drug use and new drug development [25]. Therefore, on the basis of network pharmacology, this study screened important targets related to MCI through disease databases and further identified and analyzed the corresponding compounds and herbs. By analyzing the complex network of targets, components and herbs, we explored the core components and herbs important for the treatment of MCI. The results of this study are expected to provide new ideas and a basis for the development of new therapies and drugs for MCI. The overall research idea of this study is shown in Figure 1.

## 2. Materials and Methods

### 2.1. Collection and Screening of MCI Disease Targets

We used the GeneCard [26] (https://www.genecards.org/), OMIM [27] (http://www.omim.org/), DisGeNET [28] (http://www.disgenet.org/) and DrugBank [29] databases to search and access MCI disease targets. In DisgeNet, the confidence score is determined by the number of times that the gene-disease association appears repeatedly in all data sources, reflecting the reliability of the gene-disease association [30]. The GeneCard database established a correlation ranking of genes and diseases through the Gifts algorithm [31]. Based on the relevance score, targets with a higher relevance degree can be further screened from many targets corresponding to specific diseases.

### 2.2. Acquisition of Candidate Components Corresponding to Targets

The UniProt database was used to obtain the protein names of the targets. TCMSP (http://ibts.hkbu.edu.hk/LSP/tcmsp.php) was used to obtain the targets corresponding to potential active ingredients. After setting the ADME screening conditions to oral bioavailability (OB) ≥30% and drug-like property (DL) ≥0.18 [32], the candidate ingredients were obtained. The targets and candidate components were imported into Cytoscape 3.7.2 software to construct a target-component network. We carried out a network topology analysis of the target-component network to obtain the core components and targets.

### 2.3. Herbal Acquisition and Target - Component - Herb Network Construction

Herbs with candidate ingredients were collected from the TCMSP. The names of herbal medicines are standardized and unified in accordance with the Chinese Pharmacopoeia (2020), Traditional Chinese Medicine (“Thirteenth Five-Year Plan” textbook) and Chinese Clinical Medicine Dictionary [33]. Furthermore, we obtained the characteristics of Chinese herbal medicines, including their properties, flavor, and channel tropism, to obtain frequency statistics and analyze and summarize the rules. We performed a network topological analysis based on the construction of a target-component-herb network to identify the key nodes and evaluate the efficacy of herbs and candidate components in the treatment of MCI.

### 2.4. Molecular Docking of Core Target Components

The MOL2 structure of the core components (ligand) was downloaded from the TCMSP database and saved as a “PDBQT” lattice document after setting the rotatable key through AutoDock Tools. We downloaded the core target protein structure from the PDB database (http://www.rcsb.org/) and used PyMOL 1.8 software to remove water molecules and isolate the primary ligand. After preservation, the protein structure was imported into AutoDock Tools 1.5.6 software to hydrogenate, calculate the total charge, and set the atomic type, and the file was saved in “PDBQT” format. Finally, AutoDock4 software was used for molecular docking to calculate the minimum binding energy between the active ingredients and the targets. PyMOL 2.3 and Ligplot 2.2 were used for visualization and the docking conformation analysis.

## 3. Results

### 3.1. Acquisition of Targets in MCI

Using the GeneCard database, we obtained 8019 targets related to MCI. Due to the large number of targets, we screened targets with strong relationships by using an association score greater than 0.2. Ultimately, 97 targets were acquired. After screening with a score greater than 0.02, we obtained 138 targets in the DisGeNET database. Thirty-three and 100 targets were obtained from the DrugBank database and OMIM database, respectively. After combining the targets obtained from the above four databases and eliminating duplications, in total, 190 targets were obtained. Among these 190 targets, in total, 28 underlying targets were successfully matched with compounds that met the ADME criteria as shown in Table 1.

### 3.2. Acquisition of Potential Compounds and Construction of the Target-Component Network

**Figure 2:**
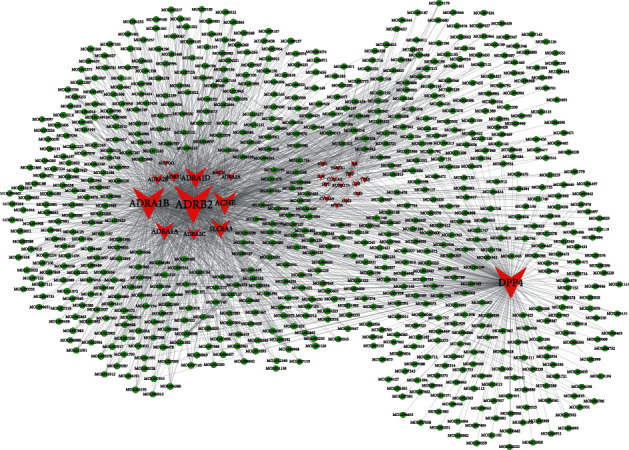
Target-component networks.

After the ADME condition screening, 492 potential compounds were identified from 28 targets in the TCMSP database. We constructed a target-component network by using 28 underlying targets and 492 potential components as shown in Figure 2. There are 745 nodes and 2026 edges in the network diagram. The red nodes in the network represent the targets, the green nodes represent the components, and the edges represent the relationships between the targets and components. The topological analysis of the network showed that the top 5 targets were ADRB2, ADRA1B, DPP4, ACHE and ADRA1D, and their corresponding degree values were 411, 329, 319, 191 and 171, respectively. Therefore, the above targets play an important role in improving cognitive impairment and are important targets for TCM intervention for mild cognitive impairment.

### 3.3. Construction of Target-Component-Herbal Networks

**Figure 3:**
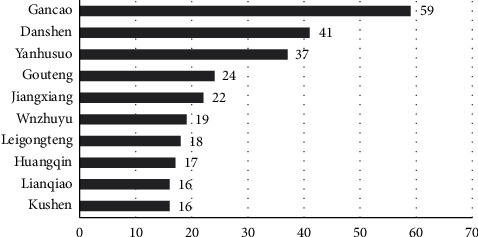
Top 10 herbs in the target-ingredient-herb network.

In total, 449 herbs containing candidate ingredients were obtained from the TCMSP database. After standardization according to the Chinese Pharmacopoeia (2020), Traditional Chinese Medicine (“Thirteenth Five-Year Plan” textbook) and Chinese Clinical Medicine Dictionary, we finally obtained 387 herbs. We constructed a target-component-herb network using Cytoscape 3.7.2 software to explore the interrelationships of the network. The target-component-herb network contains 1189 nodes with 4173 edges. The network topology analysis showed that the top 10 herbs ranked by degree were Gancao (*Glycyrrhiza uralensis* fisch), Dangshen (*Salvia miltiorrhiza* bunge), Yanhusuo (*Corydalis yanhusuo*), Gouteng (*Uncaria rhynchophylla*), Jiangxiang (*Dalbergia odorifera*), Wuzhuyu (*Tetradium ruticarpum*), Leigongteng (*Tripterygium wilfordii* hook.f.), Huangqin (*Scutellaria baicalensis* georgi), Kushen (*Sophora flavescens* aiton) and Lianqiao (*Forsythia suspensa*). As shown in Figure 3, these herbs contain 59, 41, 37, 24, 22, 19, 18, 17, 16, and 16 components. Due to the large number of components in the network, we identified the components with a strong correlation by calculating the median of their degree values. After two calculations, the median was 4 and 6, respectively. Therefore, components with degrees greater than or equal to 12 are considered potential core components in our study. The top 5 components are beta-sitosterol, quercetin, kaempferol, stigmasterol and luteolin, and the remaining components are shown in Table 2.

**Figure 4:**
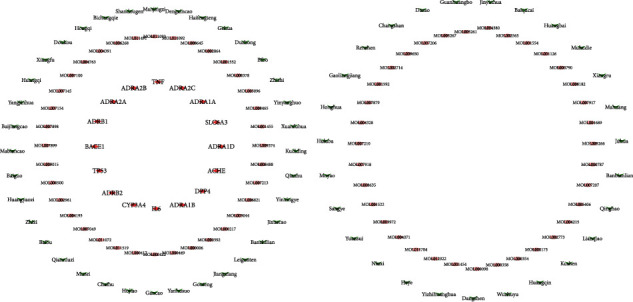
Target-component-herbal network diagram.

To more clearly show the relationship among the targets, compounds and herbs, we selected targets, compounds and herbs with a degree greater than 8 to reconstruct the target-compound-herbal network as shown in Figure 4.

**Figure 5:**
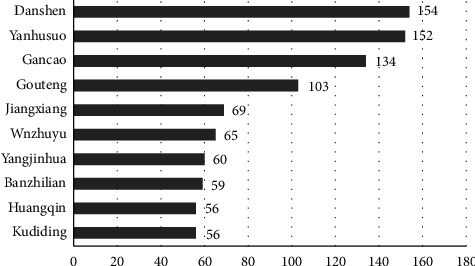
Top 10 herbs in the target-herb network.

Using ingredients as a bridge, we constructed a target-herb network to explore the relationship between targets and herbal medicine. The results of the network analysis showed that the herbs with the most targets were Danshen (*Salvia miltiorrhiza* bunge), Yanhusuo (*Corydalis yanhusuo*), Gancao (*Glycyrrhiza uralensis* fisch), Gouteng (*Uncaria rhynchophylla*), Jiangxiang (*Dalbergia odorifera*), Wuzhuyu (*Tetradium ruticarpum*), Yangjinhua (*Datura metel* L.), Banzhilian (*Scutellaria barbata* D. don), Kudiding (*Corydalis bungeana* turcz.) and Huangbai (*Phellodendron amurense* rupr.). As shown in Figure 5, their degree values are 154, 152, 134, 103, 69, 65, 60, 59, 56, and 56. Therefore, based on the results of the above network analysis, we speculate that the abovementioned herbs play a very important role in the treatment of MCI.

### 3.4. Analysis of the Properties, Flavor and Meridian Distribution of Herbal Medicine

**Figure 6:**
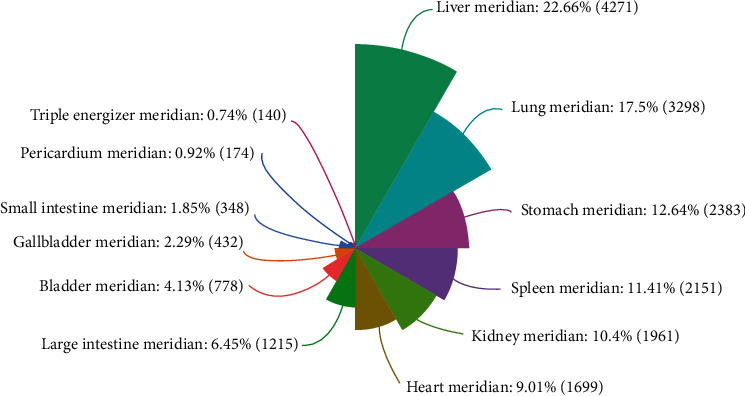
The meridian distribution of herbs.

We analyzed the properties, taste and meridian tropism of 387 herbs that could interfere with MCI. The results showed that herbs with a bitter, pungent or sweet taste accounted for 81.22%; among these herbs, bitter herbs had the highest frequency, followed by pungent and sweet herbs. Most herbs used to treat MCI are warm, cold, or mild-natured. The results of the meridian tropism analysis showed that among the 387 herbs, those belonging to the liver meridian were the most frequent, accounting for 22.66%. The proportions of herbs belonging to the lung meridian, stomach meridian and spleen meridian were 17.5%, 12.64% and 11.41%, respectively. The results are presented in Table 3 and Figure 6.

### 3.5. Molecular Docking Results

**Figure 7:**
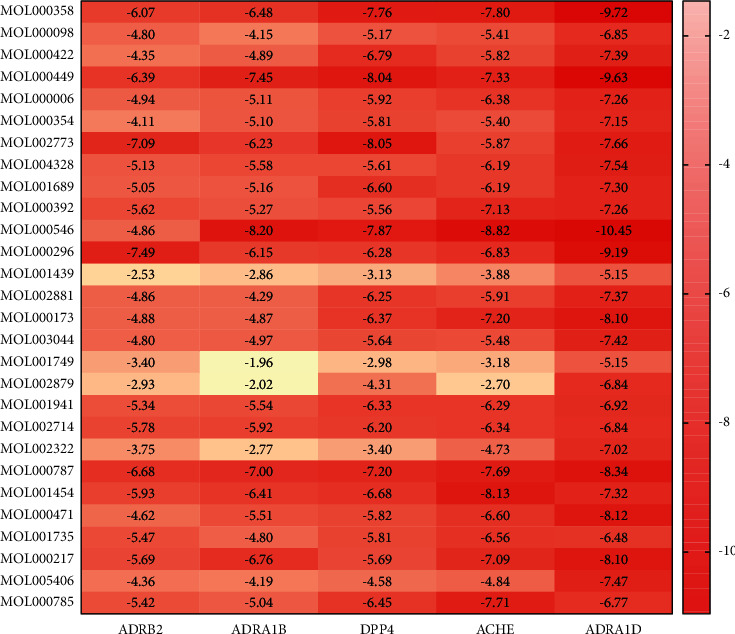
Heatmap of the molecular docking results.

In total, 140 receptor–ligand combinations were obtained by the molecular docking of 28 core components with five core targets, namely, beta-2 adrenergic receptor (ADRB2), alpha-1B adrenergic receptor (ADRA1B), dipeptidyl peptidase 4 (DPP4), acetylcholinesterase (ACHE) and alpha-1D adrenergic receptor (ADRA1D). The lower the binding energy (affinity), the better the docking effect. In general, a binding energy (affinity) less than −5.0 kcal/mol^−1^ indicates good binding activity, while a binding energy less than −7 kcal/mol^−1^ demonstrates exceptionally strong binding activity. The affinity of 65 receptor–ligand combinations was between −7 kcal/mol^−1^ and −5 kcal/mol^−1^, accounting for 46.43%. There were 39 groups with an affinity less than −7 kcal/mol^−1^, accounting for 27.68%; among these, diosgenin (MOL000546) had the lowest affinity with the ADRA1D target. Therefore, the molecular docking results showed that 74.29% of the receptor–ligand combinations had good binding ability. The molecular docking results are shown in a heatmap (Figure 7).

Of the 140 combinations, 39 combinations existed in the target-compound network. Among the 39 combinations, there were 27 groups with binding energies between −7 kcal/mol^−1^ and −5 kcal/mol^−1^ and 5 groups with binding energies less than −7 kcal/mol^−1^, indicating that most combinations had good binding activity. To some extent, the results support the reliability of the interaction between the components and targets in the target-compound network.

The molecular docking results show that there are 101 new combinations outside the target-component network. The affinity of 72 new combinations was less than −5 kcal/mol^−1^, indicating good docking activity. Among the 72 new combinations, the top 5 combinations with the strongest combination ability were ADRA1D-diosgenin (−10.45 kcal/mol^−1^), ADRA1D-beta-sitosterol (−9.72 kcal/mol^−1^), ADRA1D-stigmasterol (−9.63 kcal/mol^−1^), ADRA1D-hederagenin (−9.19 kcal/mol^−1^) and DPP4-diosgenin (−8.82 kcal/mol^−1^). The binding ability of these 5 combinations is better than that of most combinations in the target-compound network, indicating that these combinations are more likely to have strong drug-target relationships. Therefore, the docking results can provide a reference for the subsequent experimental screening and design of relevant Chinese medicines and components.

**Figure 8:**
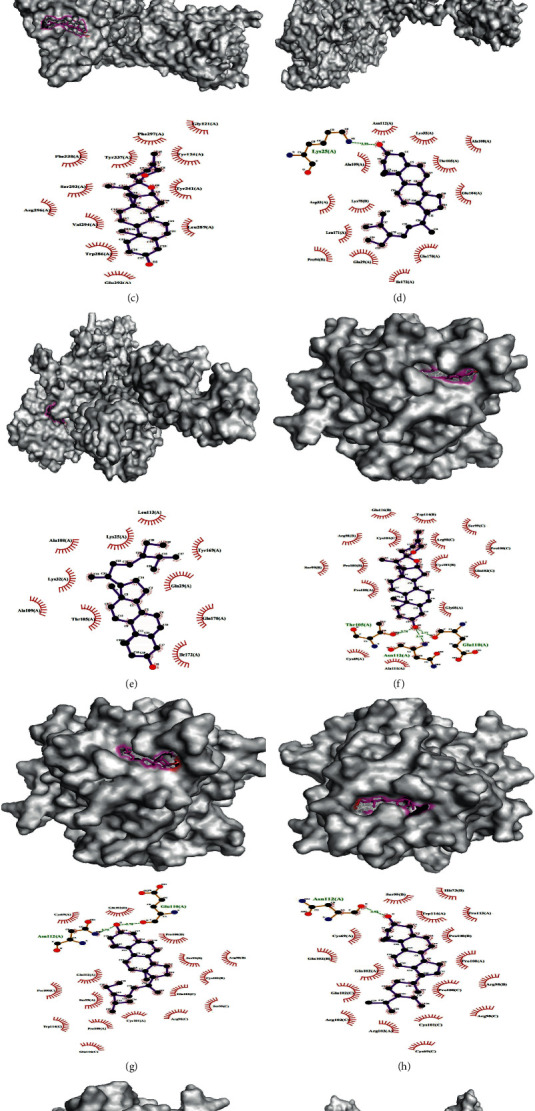
Diagram of the molecular docking patterns between the components and core targets. (a) ADRA1B-Stigmasterol; (b) DPP4-kaempferol; (c) ADRA1B-beta-sitosterol; (d) ADRB2-Stigmasterol; (e) ADRB2-beta-sitosterol; (f) ADRA1D-diosgenin; (g) ADRA1D-beta-sitosterol; (h) ADRA1D-Stigmasterol; (i) ADRA1D-hederagenin; (j) ACHE-diosgenin.

Regarding the combinations in the network, five ideal combinations were selected by considering the molecular docking affinity value and the degree of the target-composition-herb network as shown in Figures 8(a)–8(e). Regarding the combinations outside the network, we selected 5 ideal combinations according to the strength of the combination ability as shown in Figures 8(f)–8(j).

## 4. Discussion

It is of great clinical significance to explore the pathogenesis of MCI in relation to TCM and Western medicine for the prevention and treatment of dementia. There is no exact name for MCI in Chinese medicine. According to its core symptoms of memory loss, MCI can be classified as “forgetfulness” in Chinese medicine. According to the theory of TCM, the main pathogenesis of MCI is the obstruction of brain collateral, an underfilled brain marrow, mind disusing and the deactivation of mental machinery [15]. Western medicine believes that the A*β* neurotoxicity mechanism, Tau protein hyperphosphorylation mechanism, cholinergic mechanism, oxidative stress injury mechanism, cellular inflammatory factors and other mechanisms play an important role in the pathogenesis of MCI [34–36].

### 4.1. Core Targets

The results of the target-component network analysis showed that ADRB2, ADRA1B, DPP4, ACHE and ADRA1D played a key role in the treatment of mild cognitive impairment. ADRB2, also known as *β* 2-adrenergic receptor (*β*2AR), is a subtype of *β*-adrenergic receptor that is mainly distributed in the hippocampus and cortex in the brain and is associated with memory formation [37, 38]. Studies have shown that the activation of *β*2AR can affect learning and memory function by promoting various forms of long-term enhancement (LTP) [39–41]. Meanwhile, the activation of *β*2ARs can also overcome the adverse effects of A*β* on LTP [42]. Furthermore, it has been shown that blocking *β*2-AR exacerbates cognitive deficits and reduces dendrite branching in AD mice and increases A*β* accumulation by enhancing APP phosphorylation [43]. Studies have shown that *α*-1B knockout mice exhibit impaired spatial learning of novelty and exploration [44], which is closely related to the *α*-1B adrenergic receptor-mediated norepinephrine pathway. In terms of nonspatial memory function, *α*-1B knockout mice showed a decrease in short-term delay and a significant decrease in long-term delay in a passive avoidance test, suggesting that alpha (1B)-AR may be involved in the regulation of memory consolidation and fear-driven exploration [45, 46]. Dipeptidyl peptidase 4 (DPP4) is a multifunctional exopeptidase that plays a key role in GLP-1 degradation [47], inflammation [48], and oxidative stress responses [49], which are closely associated with the onset of cognitive decline [50, 51]. DPP4 inhibitors have been shown to control blood glucose levels and prevent the exacerbation of cognitive impairment in older type 2 diabetes patients with mild cognitive impairment [52]. Acetylcholinesterase (AChE) is involved in inflammatory reactions, neuronal apoptosis, oxidative stress and the aggregation of pathological proteins, which are closely related to the pathogenesis of neurodegenerative diseases [53]. Studies have shown that low doses of donepezil (2.5 mg/d) can improve cognitive function, especially memory function, in patients with aMCI [54]. ADRA1D is not involved in spatial learning and memory but plays an important role in attention and working memory [55]. The above studies indicate that the above targets play an important role in improving cognitive impairment and are the preferred targets for TCM treatment of mild cognitive impairment.

### 4.2. Core Components

By analyzing the target-composition-herbal network, the core ingredients we obtained included beta-sitosterol, quercetin, kaempferol, stigmasterol and luteolin. Beta-sitosterol, a phytosterol that can easily penetrate the blood–brain barrier and accumulate in the brain, alleviates memory and behavior deficits by inhibiting cholinesterase-mediated acetylcholine degradation [56]. The evidence suggests that *β*-sitosterol improves memory and learning disabilities and may reduce A*β* deposition in amyloid protein precursor/presenilin 1 (APP/PS1) double transgenic mice [57]. Quercetin is a flavonoid that has been shown to have a wide range of activities against a variety of diseases and disorders. Quercetin has biological effects, such as anti-apoptosis, inhibition of oxidative stress, inflammation and promotion of neurogenesis, and has potential therapeutic effects in various neurodegenerative diseases [58]. Experimental studies have shown that quercetin can improve cognitive deficits and enhance learning and memory ability, which is related to a reduction in senile plaques and improvement in mitochondrial dysfunction, and has antioxidant and anti-apoptosis properties [59, 60]. Furthermore, quercetin ameliorates cognitive impairment in aging mice by inhibiting NLRP3 inflammasome activation [61]. Kaempferol, as a flavonoid, has been recognized as having antioxidant, anti-inflammatory and antineurotoxic effects [62]. Kaempferol can improve the cognitive decline in AD mice induced by intracerebral injection of A*β*_1-42_, which may be related to a reduction in oxidative stress and enhancement in the BDNF/TrkB/CREB signaling pathway [63]. In addition, kaempferol can regulate the cholinergic system in the brain, thereby improving the memory impairment induced by scopolamine [64]. Stigmasterol, one of the most common phytosterols, has the potential to improve cognitive impairment, motor coordination, and oxidative stress in vanadium-induced neurotoxicity [65]. One study showed that stigmasterol can significantly improve scopolamine-induced memory impairment in mice, which may be mediated by the activation of estrogen or NMDA receptors to enhance the cholinergic neurotransmission system [66]. In a rat model of cognitive impairment induced by amyloid beta (A*β*) peptide, luteolin significantly improved spatial learning and working memory impairment during the Morris water maze test and single passive avoidance test, possibly due to its regulation of the cholinergic system and inhibition of oxidative damage [67]. Luteolin can improve cognitive impairment in chronic cerebral hypoperfusion rats by scavenging oxygen free radicals, enhancing the antioxidant capacity, reducing the production of lipid peroxides, and inhibiting the inflammatory response of the cerebral cortex and hippocampus induced by chronic cerebral hypoperfusion [68]. In summary, the above core ingredients can be used for the treatment of mild cognitive impairment and have the potential for further drug development.

### 4.3. Herbal Medicine

Regarding the unique characteristics of herbal medicine, namely, the performance and function of the medicine, there are four properties, five flavors and meridian tropism. The results showed that the herbal medicines with therapeutic effects on MCI were mainly bitter, spicy and sweet in taste and warm in nature. The theory of meridian tropism reflects the selective effect of herbal medicine on specific zang-fu organs or meridians of the human body, which plays a major or special therapeutic effect on lesions in these parts. In this study, meridian tropism was mainly observed in the liver meridian and lung meridian. In traditional Chinese medicine, the liver is believed to store blood, regulate the blood volume and prevent bleeding, and the lungs regulate the movement of qi. Therefore, the herbs that belong to the liver and lung channels have the effects of regulating qi movement and promoting blood circulation in the treatment of MCI.

Through the target-component-herb network analysis using components as media, it was found that the core herbs were mainly Danshen (*Salvia miltiorrhiza* bunge), Yanhusuo (*Corydalis yanhusuo*), Gancao (*Glycyrrhiza uralensis* fisch), Gouteng (*Uncaria rhynchophylla*) and Jiangxiang (*Dalbergia odorifera*). Salvia miltiorrhiza bunge, a traditional Chinese medicine commonly used for cardiovascular and cerebrovascular diseases, has attracted increasing attention in the treatment of cognitive disorders, especially AD [69]. Studies have shown that various components of Salvia miltiorrhiza bunge have a variety of pharmacological effects related to improving cognitive impairment, such as anti-inflammatory, antioxidant, anti-apoptotic, and anti-A*β* effects and the regulation of the cholinergic system [70–73]. Tan IIA plays an anti-inflammatory and neuroprotective role by inhibiting astrocyte proliferation, upregulating Akt expression, and inhibiting NF-*κ*B and caspase-3 production in an AD model [74, 75]. However, in terms of adverse reactions, studies have found that depside salt injection made from Salvia miltiorrhiza has some side effects, such as headache, head distension, dizziness, facial flushing, skin itching, etc. [76].

Corydalis yanhusuo is a traditional Chinese medicine that promotes blood circulation and removes blood stasis, and its main active ingredient is bioactive alkali [77]. Modern pharmacological studies have shown that corydalis yanhusuo can prevent acute global cerebral ischemia–reperfusion injury in rats, inhibit Ca^2+^ accumulation in cerebral ischemia–reperfusion tissue, and alleviate neurological dysfunction in rats [78, 79]. Studies have shown that the total alkaloids of Corydalis yanhusuo can improve the learning and memory ability of chronic cerebral hypoperfusion rats by alleviating neuron injury and increasing the expression of vascular endothelial growth factor [80]. However, it was reported that the total alkaloids of Corydalis yanhusuo (473.36 mg/kg) could cause liver injury, muscle tremor and renal hemorrhage in mice [81].

*Glycyrrhiza uralensis* fisch has complex ingredients, such as glycyrrhizic acid, glycyrrhetinic acid, flavonoids and other components, that have anti-inflammatory and neuroprotective effects [82, 83]. Studies have shown that glycyrrhizin can regulate a variety of anti-apoptotic and proapoptotic factors and exert anti-inflammatory effects by inhibiting the phosphorylation of HMGB1 through the ERK signaling pathway [84]. Glycyrrhizic acid can inhibit the aggregation of *β*-amyloid, scavenge free radicals, and reduce the expression of NO, TNF-*α*, IL-1*β*, Caspase3 and BAX, thereby inhibiting neuronal apoptosis and playing a neuroprotective role [85]. However, studies have found that glycyrrhizic acid and glycyrrhetinic acid have pseudaldosterone effects, which can cause hypertension, edema and other adverse reactions [86].

Uncaria rhynchophylla has a variety of pharmacological effects on the central nervous system, such as cerebral ischemia and hypoxia, neurodegenerative diseases and other neuroprotective effects [87]. Uncarine, one of the main components of Uncaria rhynchophylla, inhibits the neurotoxicity induced by soluble A*β*1-42 by inhibiting the overactivation of extracsynaptic NMDA receptors containing NR2B and plays a neuroprotective role [88]. Another study showed that uncarine significantly improved hippocampal synaptic function in mouse models of AD, which was related to blocking epHA4-dependent signaling in hippocampal neurons and decreasing EphA4 activity in the mouse hippocampus [89]. Studies have shown that uncarine is safe at low concentrations (less than 400 mol/L) but neurotoxic at high concentrations, which may be associated with nonselective action on NMDA receptors [90, 91]. Furthermore, isuncarine inhibited the formation of nerve fibers and tangles caused by A*β* breakdown in AD mice and altered their cognitive and memory deficits [92].

It was found that (2R, 3R)-obtusafuran, one of the components of Dalbergia odorifera, had anti-neuroinflammatory effects, which inhibited the expression of the iNOS protein, the release of NO, COX-2 and PGE2, and the synthesis of TNF-*α* and IL-1*β* in LPS-induced mouse microglial BV2 cells [93]. The above core herbs all act on multiple MCI-related targets and have the potential to improve cognitive dysfunction. These findings not only prove the importance of core herbs in the treatment of MCI but also provide a new approach for the treatment of MCI by traditional Chinese medicine. Therefore, on the basis of ingredients as intermediates, searching for herbs with multiple targets may be another strategy for exploring treatments for MCI.

In conclusion, based on network pharmacology, our study screened MCI-related targets through multiple databases and matched corresponding components and herbs using the TCMSP platform to build a complex network of target-component herbs. By performing a topological analysis of the network, we obtained the core targets (ADRB2, ADRA1B, DPP4, ACHE and ADRA1D) that may contribute to the treatment of MCI. Based on the core targets, we further screened the core ingredients (beta-sitosterol, quercetin, kaempferol, stigmasterol and luteolin) and important herbs (Danshen, Yanhusuo, Gancao, Gouteng and Jiangxiang) for the treatment of MCI. Using molecular docking technology, we preliminarily verified the in-network binding activity and out-of-network connection reliability of the core targets and core components. In addition, the core ingredients and herbs identified in this study could provide a certain basis for the exploration of the prescription of integrated traditional Chinese and Western medicine for MCI treatment.

However, due to the limitations of network pharmacology, the data sources and analysis mostly rely on specific databases, and the database information is mainly derived from the published literature; thus, there is a certain bias in the collection of the database information, which may affect the integrity and accuracy of the results of this study, leading to research results bias. Another limitation of this study is the lack of experimental validation; thus, further biological confirmation is necessary.

## 5. Conclusion

In this study, we used web-based pharmacology techniques to find important MCI-related targets by searching and screening multiple databases. On this basis, we matched potential ingredients and herbs using the TCMSP platform. We further discussed and analyzed the core components, herbs and related mechanisms of TCM in the treatment of MCI by constructing a target-component-herb network. The results of this study can effectively and systematically screen herbal and compound components for the treatment of MCI and provide a reference for further experimental research, thereby reducing the economic cost of drug development and research investigating the treatment of MCI.

## Figures and Tables

**Table 1:** Information of potential MCI-related targets.

	Gene symbol	Uniprot ID	Protein name
1	IL6	P05231	Interleukin-6
2	BACE1	P56817	Beta-secretase 1
3	IL1B	P01584	Interleukin-1 beta
4	TNF	P01375	Tumor necrosis factor
5	ADIPOQ	Q15848	Adiponectin
6	RUNX1T1	Q06455	Protein CBFA2T1
7	ACHE	P22303	Acetylcholinesterase
8	SOD1	P00441	Superoxide dismutase [Cu–Zn]
9	SLC6A3	Q01959	Sodium-dependent dopamine transporter
10	HMOX1	P09601	Heme oxygenase 1
11	IL10	P22301	Interleukin-10
12	MTOR	P42345	Serine/threonine-protein kinase mTOR
13	HTT	P42858	Huntingtin
14	NOS3	P29474	Nitric oxide synthase, endothelial
15	COL1A2	P08123	Collagen alpha-2(I) chain
16	GLB1	P16278	Beta-galactosidase
17	DPP4	P27487	Dipeptidy peptidase 4
18	TP53	P04637	Cellular tumor antigen p53
19	VEGFA	P15692	Vascular endothelial growth factor A
20	ADRB1	P08588	Beta-1 adrenergic receptor
21	ADRB2	P07550	Beta-2 adrenergic receptor
22	ADRA1A	P35348	Alpha-1A adrenergic receptor
23	CYP3A4	P08684	Cytochrome P450 3A4
24	ADRA2C	P18825	Alpha-2C adrenergic receptor
25	ADRA2B	P18089	Alpha-2B adrenergic receptor
26	ADRA2A	P08913	Alpha-2A adrenergic receptor
27	ADRA1D	P25100	Alpha-1D adrenergic receptor
28	ADRA1B	P35368	Alpha-1B adrenergic receptor

**Table 2:** Information of the potential core components (degree ≥ 12).

Mol ID	Mol name	Degree	OB	DL
MOL000358	Beta-sitosterol	246	36.91	0.75
MOL000098	Quercetin	201	46.43	0.28
MOL000422	Kaempferol	139	41.88	0.24
MOL000449	Stigmasterol	138	43.83	0.76
MOL000006	Luteolin	98	36.16	0.25
MOL000354	Isorhamnetin	42	49.60	0.31
MOL002773	Beta-carotene	31	37.18	0.58
MOL004328	Naringenin	24	59.30	0.21
MOL001689	Acacetin	24	34.97	0.24
MOL000392	Formononetin	23	69.67	0.21
MOL000546	Diosgenin	18	80.88	0.81
MOL000296	Hederagenin	17	36.91	0.75
MOL001439	Arachidonic acid	16	45.57	0.20
MOL002881	Diosmetin	16	31.14	0.27
MOL000173	Wogonin	15	30.68	0.23
MOL003044	Chryseriol	15	35.85	0.27
MOL001749	zinc03860434	15	43.59	0.35
MOL002879	Diop	15	43.59	0.39
MOL001941	Ammidin	14	34.55	0.22
MOL002714	Baicalein	14	33.52	0.20
MOL002322	Isovitexin	13	31.29	0.71
MOL000787	Fumarine	13	59.26	0.83
MOL001454	Berberine	13	36.86	0.78
MOL000471	Aloe-emodin	12	83.38	0.24
MOL001735	Dinatin	12	30.97	0.27
MOL000217	(s)-Scoulerine	12	32.28	0.54
MOL005406	Atropine	12	45.97	0.19
MOL000785	Palmatine	12	64.60	0.65

**Table 3:** The properties and taste of herbs.

Flavor	Frequency	Proportion (%)	Properties	Frequency	Proportion (%)
Bitter	4140	33.47	Warm	1975	24.90
Pungent	3154	25.50	Cold	1806	22.77
Sweet	2752	22.25	Mild-natured	1514	19.09
Astringent	643	5.20	Slight cold	1152	14.52
Slightly bitter	576	4.66	Cool	754	9.50
Sour	537	4.34	Slight warm	494	6.23
Salty	222	1.79	Hot	208	2.62
Light	173	1.40	Great cold	25	0.32
Slightly pungent	80	0.65	Great hot	4	0.05
Slightly sweet	65	0.52			
Slightly sour	28	0.22			

## Data Availability

Supporting data for the results of this study are included in the article and supplementary Materials.

## Supplementary Materials

Supplementary Materials Figure 7 data: Heatmap of the molecular docking results.
