# Supplementary material for: Medication Rules in Herbal Medicine for Mild Cognitive Impairment: A Network Pharmacology and Data Mining Study
Source: Evid Based Complement Alternat Med. 2022 May 18;2022:2478940. doi: 10.1155/2022/2478940 (PMC9132671; doi:10.1155/2022/2478940)
Supplement: Supplementary Materials — Figure 7 data: Heatmap of the molecular docking results. [file 2478940.f1.doc]

**SUPPLEMENTARY DESCRIPTION**

**Supplementary Material**

**Figure 7 data**: Heatmap of the molecular docking results.

| MolID | MolName | ADRB2 | ADRA1B | DPP4 | ACHE | ADRA1D |
| --- | --- | --- | --- | --- | --- | --- |
| MOL000358 | beta-sitosterol | -6.07 | -6.48 | -7.76 | -7.8 | -9.72 |
| MOL000098 | quercetin | -4.8 | -4.15 | -5.17 | -5.41 | -6.85 |
| MOL000422 | kaempferol | -4.35 | -4.89 | -6.79 | -5.82 | -7.39 |
| MOL000449 | Stigmasterol | -6.39 | -7.45 | -8.04 | -7.33 | -9.63 |
| MOL000006 | luteolin | -4.94 | -5.11 | -5.92 | -6.38 | -7.26 |
| MOL000354 | isorhamnetin | -4.11 | -5.1 | -5.81 | -5.4 | -7.15 |
| MOL002773 | beta-carotene | -7.09 | -6.23 | -8.05 | -5.87 | -7.66 |
| MOL004328 | naringenin | -5.13 | -5.58 | -5.61 | -6.19 | -7.54 |
| MOL001689 | acacetin | -5.05 | -5.16 | -6.6 | -6.19 | -7.3 |
| MOL000392 | formononetin | -5.62 | -5.27 | -5.56 | -7.13 | -7.26 |
| MOL000546 | diosgenin | -4.86 | -8.2 | -7.87 | -8.82 | -10.45 |
| MOL000296 | hederagenin | -7.49 | -6.15 | -6.28 | -6.83 | -9.19 |
| MOL001439 | arachidonic acid | -2.53 | -2.86 | -3.13 | -3.88 | -5.15 |
| MOL002881 | Diosmetin | -4.86 | -4.29 | -6.25 | -5.91 | 7.37 |
| MOL000173 | wogonin | -4.88 | -4.87 | -6.37 | -7.2 | -8.1 |
| MOL003044 | Chryseriol | -4.8 | -4.97 | -5.64 | -5.48 | -7.42 |
| MOL001749 | ZINC03860434 | -3.4 | -1.96 | -2.98 | -3.18 | -5.15 |
| MOL002879 | Diop | -2.93 | -2.02 | -4.31 | -2.7 | -6.84 |
| MOL001941 | Ammidin | -5.34 | -5.54 | -6.33 | -6.29 | -6.92 |
| MOL002714 | baicalein | -5.78 | -5.92 | -6.2 | -6.34 | -6.84 |
| MOL002322 | isovitexin | -3.75 | -2.77 | -3.4 | -4.73 | -7.02 |
| MOL000787 | Fumarine | -6.68 | -7 | -7.2 | -7.69 | -8.34 |
| MOL001454 | berberine | -5.93 | -6.41 | -6.68 | -8.13 | -7.32 |
| MOL000471 | aloe-emodin | -4.62 | -5.51 | -5.82 | -6.6 | -8.12 |
| MOL001735 | Dinatin | -5.47 | -4.8 | -5.81 | -6.56 | -6.48 |
| MOL000217 | (S)-Scoulerine | -5.69 | -6.76 | -5.69 | -7.09 | -8.1 |
| MOL005406 | atropine | -4.36 | -4.19 | -4.58 | -4.84 | -7.47 |
| MOL000785 | palmatine | -5.42 | -5.04 | -6.45 | -7.71 | -6.77 |
